# Impact of COVID-19 pandemic on marriage, divorce, birth, and death in Kerman province, the ninth most populous province of Iran

**DOI:** 10.1038/s41598-024-54679-5

**Published:** 2024-02-17

**Authors:** Shiva Pouradeli, Hassan Ahmadinia, Mohsen Rezaeian

**Affiliations:** 1 Occupational Environment Research Center, Medical School, Rafsanjan University of Medical Sciences, Kerman, Iran; 2 Social Determinants of Health Research Center, Institute for Futures Studies in Health, Kerman University of Medical Sciences, Kerman, Iran; 3 Department of Epidemiology and Biostatistics, School of Health, Occupational Environment Research Center, Rafsanjan University of Medical Sciences, Rafsanjan, Iran

**Keywords:** COVID-19, Pandemics, Marriage, Divorce, Birth rate, Death rate, Iran, Public health, Population screening

## Abstract

This study examined the impact of the COVID-19 pandemic on marriage, divorce, birth, and death rates using the Poisson regression model and an interrupted time-series Poisson regression model. Before the pandemic, marriage and birth rates were decreasing, while divorce and death rates were increasing, with only the trend in birth rates being statistically significant. The immediate effect of the pandemic was a significant decrease in the divorce rate, but there were non-significant effects on birth and marriage rates. However, in the months following the onset of the pandemic, there was a statistically significant sustained effect on increasing death and divorce rates. Forecasts based on pre-pandemic data showed that by the end of 2020, marriage, divorce, death, and birth rates were higher compared to pre-pandemic levels. In conclusion, the pandemic has greatly impacted society, particularly in terms of death and divorce rates. Birth rates were not immediately affected to the time lag between decisions and actual births. Fear of COVID-19 may have increased death rates as people avoided seeking medical help. Vaccination and effective treatment strategies are vital in reducing the pandemic's impact on mortality. Supporting families financially is important due to the role of economic issues in couples’ decisions.

## Introduction

The COVID-19 pandemic started in December 2019, and rapidly spread worldwide^1^. Within a span of approximately 10 months, it infected over 33 million people and caused over a million deaths in 216 countries and regions^2^. The World Health Organization (WHO) declared it a global pandemic on March 11, 2020^3^. To control the disease, crucial measures such as limiting contact between individuals and implementing quarantine protocols were put in place^4^. Quarantine can have a significant impact on one's daily life, as it limits the ability to interact with friends, family, and society, ultimately affecting both social and individual aspects of life^5,6^. The pandemic had significant adverse effects on societies, economies, and employment rates, resulting in social and economic crises^7,8^.

Similar to the 1918 influenza pandemic, the COVID-19 pandemic had a substantial impact on marriage, divorce, and fertility rates, as well as various aspects of society, including domestic violence, conflict within families^9,10^. In the United States, for example, marriage rates initially declined in several states but later saw an increase in some areas. Divorce rates followed a similar pattern, initially decreasing and subsequently rising in certain states^11^. In Florida, Hawaii, Dallas, and Seattle, the cumulative number of marriages decreased in 2020 compared to the previous year^12^. In Japan, the pandemic led to a decline in both marriages and divorces, particularly during the state of emergency^9^. In Italy, people preferred unregistered cohabitation and marriage during the pandemic^13^. In Denmark, the rate of divorce decreased in 2020 compared to previous years^14^. Furthermore, the pandemic impacted the fertility rate^15^. Before the pandemic, Japan already faced a concerning decline in marriage and fertility rates. The birth rate declined even further in the six months following the outbreak^9^. Following the lockdown, there was a significant decrease of 20% in live births in New York, compared to the previous year^16^. Italy also experienced a decrease in the birth rate during the pandemic^17^, and the fertility intention of women in China decreased as well^18^. Conversely, the death rate increased due to the pandemic. Iran recorded 58,900 excess deaths in 31 provinces during the first nine months after the pandemic^19^, while the United States saw an additional 198,081 deaths during the pandemic^20^. In Colombia, 65% of the additional deaths in the initial weeks of the pandemic were attributed to COVID-19^21^.

Marriage, divorce, death, and birth rates are crucial factors for planning and policymaking regarding population, economy, society, and infrastructure worldwide^22^. Additionally, migration, death, birth, and fertility rate all impact population growth^23^. Iran, which had a population of 79 million in 2015 and is the 17th most populous country in the world, is projected to experience a population decrease to 70 million by 2100^24^. Iran has entered a Second Demographic Transition characterized by low fertility rates and a shift away from traditional familial values, similar to the one that occurred in Europe and North America and is now spreading to Asia^25^. The country is experiencing a cultural shift towards individualistic norms and values that prioritize personal choice over traditional family values, reflected in the growing acceptance of alternative family structures such as single-parent households, divorce, and childless families^26^. Iran has also experienced a decline in crude marriage rates and the highest growth rate of divorce among Islamic countries in the Middle East and North Africa region^27,28^. Factors such as delayed marriage, non-marriage and divorce contribute significantly to the decline in the fertility rates^29,30^. Birth rate and total fertility rate (TFR) are critical determinants of population size and composition^31^. The decrease in fertility and mortality is one of the important indicators of Iran's aging population^24^. Population aging is expected to have several negative effects, including an increase in chronic diseases^32^, a decrease in the labor force, and adverse consequences for economic development^33^. In addition to these challenges faced by all aging societies, Iran must also contend with severe internal economic issues such as poverty, inequality, high youth unemployment rates, and political and cultural conflicts^24^.

Comprehensive investigation of the relationship between population growth indicators, such as marriage, divorce, birth, and death rates, and their potential impact from the pandemic is crucial in Iran. However, there is a lack of comprehensive studies on this issue. To address this gap, our study focuses on Kerman province, the largest and ninth most populated province in southeast Iran^34^. The primary objective of this study is to analyze the impact of the COVID-19 pandemic on marriage, divorce, birth, and death rates in Kerman province, Iran.

## Method

This ecological study was conducted as part of a Ph.D. thesis. The statistical population included people who lived in Kerman Province from 2017 to 2020. Kerman Province had 23 counties with population of 3,164,718 people based on the 2016 national census^35^.

The data on marriage, divorce, birth and death was collected from the database of National Organization for Civil Registration in Kerman province from 2017 to 2020. This organization is responsible for a range of tasks, including birth and death registration, marriage and divorce registration^36^. Natural population growth, sex ratio at birth and sex ratio at dead were calculated based on birth and death data.

### Statistical methods

#### Poisson regression model

The Poisson regression model was used to estimate the incidence rate ratio (IRR) of marriages, divorces, births, and deaths (with a 95% CI) in Kerman province. Poisson regression is a generalized linear model form of regression analysis used to model count data^37^. It is important to assess the assumption of overdispersion to ensure the appropriateness of the model. To evaluate this, we examined the "Value/df" index for the "Pearson Chi-Square" in each model. The index values close to 1 indicate that there is a condition of equality between the mean and variance, suggesting that the Poisson regression model is well-fitted to the data in our study. The year 2020 (the first year of the COVID-19 epidemic) was used as a reference year, and the IRR of the other years was compared to year the 2020. Data analysis was performed using SPSS software (version 24). The significance level was considered at 0.05.

#### Interrupted time-series (ITS) analysis

To model monthly changes in marriage, divorce, birth, and death before (2017–2019) and after the COVID-19 pandemic (2020), the interrupted time-series Poisson regression model was performed. ITS analysis is a method of statistical analysis to evaluate the longitudinal effects of interventions^38^. This method is widely used for modeling the count time series^39^. In this model, the IRR's role in determining the effects of COVID-19 on variables. The IRR of the trend shows the changes in the variables in the months leading up to the pandemic, revealing how much they changed on a monthly basis before the pandemic's onset. The IRR of COVID-19 shows the immediate effect of the pandemic on the variables, indicating how much they changed at the beginning of the pandemic. The IRR index is the interaction effect of COVID-19 and the trend, revealing the sustained effect of the pandemic on the variables. It is obtained from the difference in the slope of the regression line after and before the pandemic, indicating how the trend of changes in the variables in the months following the onset of the pandemic has changed compared to before.

Data analysis was performed using R software version R 4.1.0.

#### Date

After the first confirmed case of COVID-19 was reported on February 2019 in Iran, the number of infected people increased rapidly and this disease led to a national crisis. Thus, The Iranian authorities performed several instructions of quarantines to prevent the spread of this disease^40^. The study considered March 20, 2020, as the official quarantine date and start of the pandemic time in Iran. In the first year of the pandemic (1399 SH, 2020), there were 2,440,725 new cases and 584,340 deaths reported worldwide, with Iran recording 1,786,259 new cases and 61,649 deaths^41^. The data were separated by year according to the Solar Hijri calendar (SH) from 1396 to 1399 SH^42^. As the Gregorian calendar is used in most parts of the world, the months in the solar calendar and the Gregorian calendar in this study were matched as shown in Table 1:

**Table 1:** Matching years in the solar calendar and the Gregorian calendar.

Gregorian calendar	Solar Hijri calendar	Study period
21 March 2017–20 March 2018	1396 SH	2017
21 March 2018–20 March 2019	1397 SH	2018
21 March 2019–19 March 2020	1398 SH	2019
20 March 2020–20 March 2021	1399 SH	2020

### Ethics approval

This ecological study survey was approved by the Research Ethics Committee of Rafsanjan University of Medical Sciences (IR.RUMS.REC.1400.013).

## Results

A total of 91,025 marriages, 23,072 divorces, 210,575 births, and 58,570 deaths were registered in Kerman Province from 2017 to 2020. The highest frequency of marriage and divorce was found in 2017 and 2018, respectively. The average age of marriage was 28.15 ± 1.45 years for men and 23.21 ± 1.65 years for women. The average age of divorce was 35.32 ± 2.19 years for men and 30.54 ± 2.36 years for women. The frequency of birth has decreased in both sexes from 2017 to 2020. The frequency of death has increased in women from 2017 to 2020 and in men from 2018 to 2020. The frequency of birth and death has been higher in men than in women in all years (Table 2).

**Table 2:** Frequency (percentage) of marriage, divorce, birth, and death (2017–2020).

Variables	Sex	Frequency (percentage)				
		2017	2018	2019	2020	Total
Marriage	Total	23,975 (26.34)	21,856 (24.01)	21,467 (23.58)	23,727 (26.07)	91,025 (100)
Divorce		5701 (24.71)	5969 (25.87)	5698 (24.70)	5704 (24.72)	23,072 (100)
Birth	Male	30,656 (28.42)	28,623 (26.53)	25,170 (23.33)	23,429 (21.72)	107,878 (100)
	Female	29,067 (28.30)	27,105 (26.39)	24,017 (23.39)	22,508 (21.92)	102,697 (100)
	Total	59,723 (28.36)	55,728 (26.46)	49,187 (23.36)	45,937 (21.82)	210,575 (100)
Death	Male	7787 (23.53)	7457 (22.53)	7967 (24.08)	9880 (29.86)	33,091 (100)
	Female	5761 (22.61)	5835 (22.90)	6007 (23.58)	7876 (30.91)	25,479 (100)
	Total	13,548 (23.13)	13,292 (22.69)	13,974 (23.86)	17,756 (30.32)	58,570 (100)

The natural growth of the population has dwindled over the past four years. In 2020, there was a reduction in the sex ratio at birth among males, while the sex ratio at death increased for the same gender compared to previous years. In 2020, for every 100 female births, around 104 male births were recorded. Moreover, for every 100 female deaths, approximately 80 men passed away (Table 3).

**Table 3:** Population indices from 2017 to 2020.

Population indices	2017	2018	2019	2020
Crude birth rate (Number of live births/Total population) × 1000)	19	18	16	15
Crude death rate (Number of live births/Total population) × 1000)	2	2	3	3
Natural population growth (Crude birth rate–Crude death rate)	16	15	13	11
Sex ratio at birth (Number of male births/Number of female births) × 100	105	106	105	104
Sex ratio at dead (Number of male dead/Number of female dead) × 100	74	78	75	80

The frequency of marriage and divorce in urban and rural areas increased in 2020 compared to the previous year (Table 4).

**Table 4:** Frequency of marriage and divorce by place of residence in Kerman province (2017–2020).

	2017		2018		2019		2020	
	City	Village	City	Village	City	Village	City	Village
Marriage	22,559	1416	20,017	1839	19,800	1667	21,989	1738
Divorce	5542	159	5800	169	5532	166	5535	169

The Poisson regression model was used to compare the incidence rate ratios (IRR) of variables in 2020, which marked the onset of the COVID-19 epidemic, with those observed in the preceding years. The IRR of marriage in 2020 was 1% (IRR: 1.01; 95% CI 0.992–1.029; P-value = 0.25) lower than in 2017, and 7.9% (IRR: 0.921; 95% CI 0.904–0.938) and 9.5% (IRR: 0.905; 95% CI 0.888–0.922) higher compared to 2018 and 2019, respectively (P < 0.001). The IRR of divorce in 2020 was 0.1% (IRR: 0.999; 95% CI 0.963–1.037; P-value = 0.978) higher than in 2017, 4.6% (IRR: 1.046; 95% CI 1.009–1.085; P-value = 0.014) lower than in 2018, and 0.1% (IRR: 0.999; 95% CI 0.963–1.036; P-value = 0.955) higher than in 2019.

**Figure 1:**
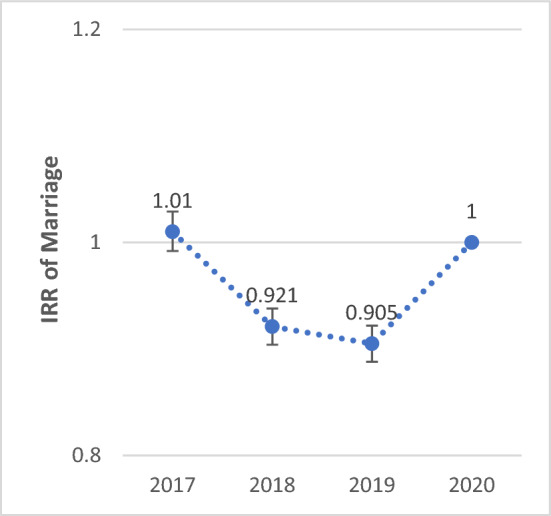
Total IRR of marriage from 2017 to 2020.

**Figure 2:**
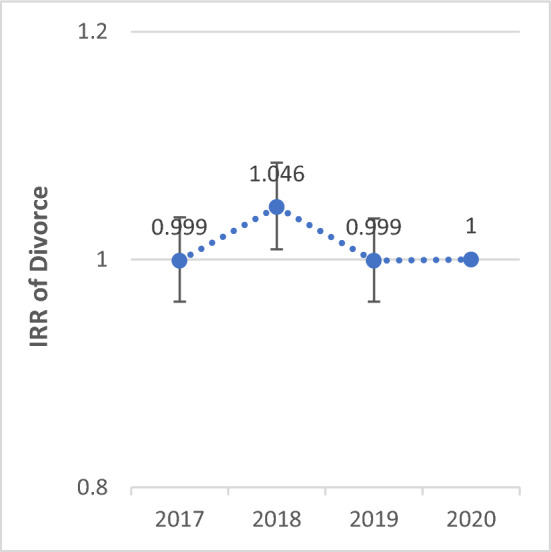
Total IRR of divorce from 2017 to 2020.

**Figure 3:**
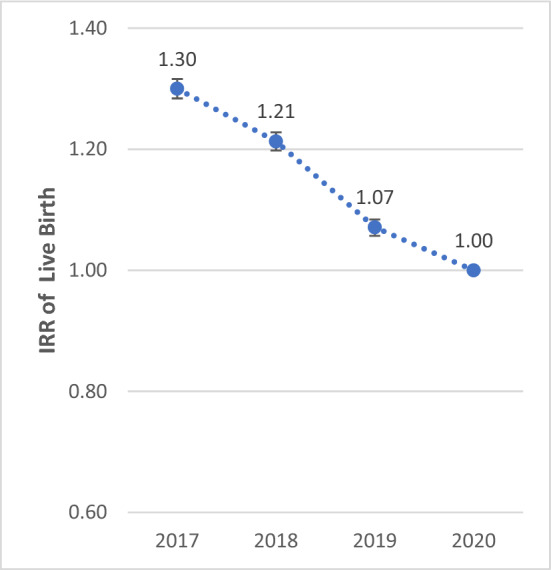
Total IRR of birth from 2017 to 2020.

**Figure 4:**
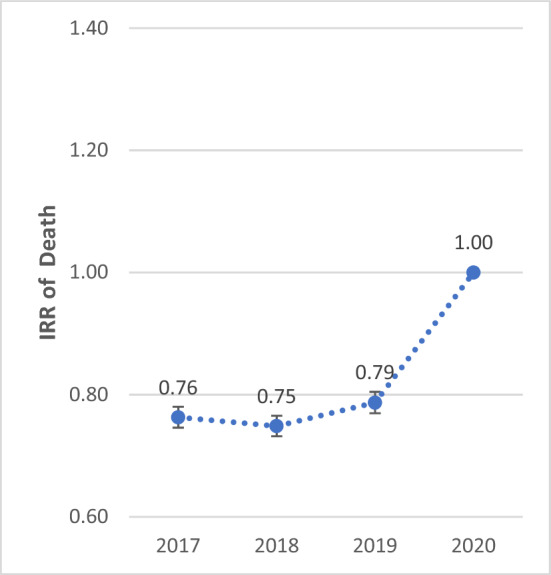
Total IRR of death from 2017 to 2020.

The IRR of birth showed a significant decreasing trend from 2017 to 2020. The IRR of birth in 2020 was 30.0% (IRR: 1.300; 95% CI 1.284–1.316), 21.3% (IRR: 1.213; 95% CI 1.198–1.228), and 7.1% (IRR: 1.071; 95% CI 1.057–1.084) lower compared to 2017, 2018, and 2019, respectively (P < 0.001). The IRR of death showed a significant increasing trend from 2017 to 2020. The IRR of death in 2020 was 23.7% (IRR: 0.763; 95% CI 0.746–0.780), 25.1% (IRR: 0.749; 95% CI 0.732–0.766), and 21.3% (IRR: 0.787; 95% CI 0.770–0.805) higher compared to 2017, 2018, and 2019, respectively (P < 0.001) (Figs. 1, 2, 3 and 4).

According to our data, prior to the pandemic, approximately 35 months ago, the average monthly number of marriages stood at 1880.114 ± 768.906. However, 13 months after the pandemic, this figure rose to 1940.076 ± 937.735. Similarly, the average monthly number of divorces experienced a decline from 482.285 ± 76.225, 35 months before the pandemic to 476.307 ± 101.386 after a span of 13 months. In terms of births, 35 months prior to the pandemic, the average stood at 4602.685 ± 436.612. However, 13 months after the pandemic, this number decreased to 3806.230 ± 242.621.Lastly, the average monthly number of deaths demonstrated an increase after the pandemic. 35 months before the pandemic, the average was 1132.314 ± 133.138. However, 13 months after the pandemic, this figure rose to 1454.307 ± 299.771 (Table 5).

**Table 5:** Monthly average of marriage, divorce, birth, and death before and after the COVID-19 pandemic.

Variable	Period	Month	Mean	SD	Median	IQR	Min	Max
Marriage	Before	35	1880.114	768.906	1945.0	887.0	399.0	3856.0
	After	13	1940.076	937.735	1962.0	929.0	567.0	4081.0
Divorce	Before	35	482.285	76.225	498.0	55.50	262.0	638.0
	After	13	476.307	101.386	488.0	50.0	184.0	626.0
Live birth	Before	35	4602.685	436.612	4708.0	708.0	3802.0	5260.0
	After	13	3806.230	242.621	3858.0	337.0	3513.0	4367.0
Death	Before	35	1132.314	133.138	1098.0	178.50	894.0	1474.0
	After	13	1454.307	299.771	1420.0	396.0	1054.0	2029.0

The marriage rate had an almost constant trend before the pandemic, with a monthly decrease of 0.9%, but this decrease was not statistically significant (IRR = 0.991; P = 0.200). At the beginning of the pandemic, there was an 18% increase in the marriage rate, but this increase was not statistically significant (IRR = 1.184; P = 0.562). The trend of changes in the marriage rate in the months following the onset of the pandemic increased by 1% compared to before the pandemic, but this increase was not statistically significant (IRR = 1.010; P = 0.755). The changes in the divorce rate had an almost constant trend before the pandemic, with a monthly increase of 0.2%, but this increase was not statistically significant (IRR = 1.002; P = 0.496). At the beginning of the pandemic, the divorce rate was a significant decrease of 23% (IRR = 0.769; P = 0.028). The trend of changes in the divorce rate in the months following the onset of the pandemic has significantly increased by 2.6% compared to before the pandemic (IRR = 1.026; P = 0.048).

The changes in the birth rate were significantly decreasing before the pandemic, with a monthly decrease of 0.8% (IRR = 0.992; P < 0.001). At the beginning of the pandemic, the immediate effect was a decrease in the birth rate by 5.2%, but this decrease was not statistically significant (IRR = 0.948; P = 0.205). The trend of changes in the birth rate in the months following the onset of the pandemic has increased by 0.7% compared to before the pandemic. However, this increase was not statistically significant (IRR = 1.007; P = 0.164). The changes in the death rate were almost constant before the pandemic, with a monthly increase of 0.3%, but this increase was not statistically significant (IRR = 1.003; P = 0.304). At the beginning of the pandemic, the immediate effect was an increase in the death rate by 1.2%, but this increase was not statistically significant (IRR = 1.012; P = 0.894). The trend of changes in the death rate in the months following the onset of the pandemic has statistically increased by 2.2% compared to before the pandemic (IRR = 1.022; P = 0.025) (Table 6).

**Table 6:** Results of the ITS Poisson regression for the whole group modeling of the COVID-19 pandemic, the trend, and the interaction between the COVID-19 pandemic and the trend.

Variable		Estimate	Std err	z	IRR	CI for IRR		P-value
						2·50%	97.5%	
Marriage	Intercept	− 7.289	0.144	− 50.669	0.001	0.001	0.001	< 0.001
	COVID-19 pandemic	0.169	0.291	0.581	1.184	0.669	2.095	0.562
	Trend (1–48 months)	− 0.009	0.007	− 1.282	0.991	0.977	1.005	0.200
	Interaction of COVID-19 pandemic and trend	0.010	0.032	0.312	1.010	0.948	1.076	0.755
Divorce	Intercept	− 8.849	0.060	− 148.116	0.000	0.000	0.000	< 0.001
	COVID-19 pandemic	− 0.263	0.120	− 2.197	0.769	0.608	0.972	0.028
	Trend (1–48 months)	0.002	0.003	0.681	1.002	0.996	1.008	0.496
	Interaction of COVID-19 pandemic and trend	0.026	0.013	1.967	1.026	1.000	1.033	0.048
Birth	Intercept	− 6.411	0.019	− 333.068	0.002	0.002	0.002	< 0.001
	COVID-19 pandemic	− 0.053	0.042	− 1.268	0.948	0.873	1.030	0.205
	Trend (1–48 months)	− 0.008	0.001	− 8.590	0.992	0.990	0.994	< 0.001
	Interaction of COVID-19 pandemic and trend	0.007	0.005	1.391	1.007	0.997	1.016	0.164
Death	Intercept	− 8.006	0.051	− 156.088	0.000	0.000	0.000	< 0.001
	COVID-19 pandemic	0.012	0.092	0.133	1.012	0.845	1.213	0.894
	Trend (1–48 months)	0.003	0.002	1.028	1.003	0.998	1.007	0.304
	Interaction of COVID-19 pandemic and trend	0.022	0.010	2.240	1.022	1.003	1.043	0.025

The incidence rate ratio (IRR) of COVID-19 represents the immediate effect of the COVID-19 pandemic on the rates of variables.

The IRR of the trend reflects the changes in variable rates in the months preceding the COVID-19 pandemic.

The IRR of the interaction between COVID-19 and the trend indicates a sustained effect of the COVID-19 pandemic on the rates.

The data presented shows that the trend of marriage was decreasing before the pandemic, but an immediate increasing effect was observed at the beginning of the pandemic. This was followed by a sustained increase with a slight slope. However, by the end of 2020, the trend of marriages was higher than predicted based on pre-pandemic data. The trend of divorce was slightly increasing before the pandemic, but an immediate decreasing effect was observed at the beginning of the pandemic. This was followed by a sustained increase with a sharp slope. However, by the end of 2020, the trend of divorces was higher than predicted based on pre-pandemic data.

**Figure 5:**
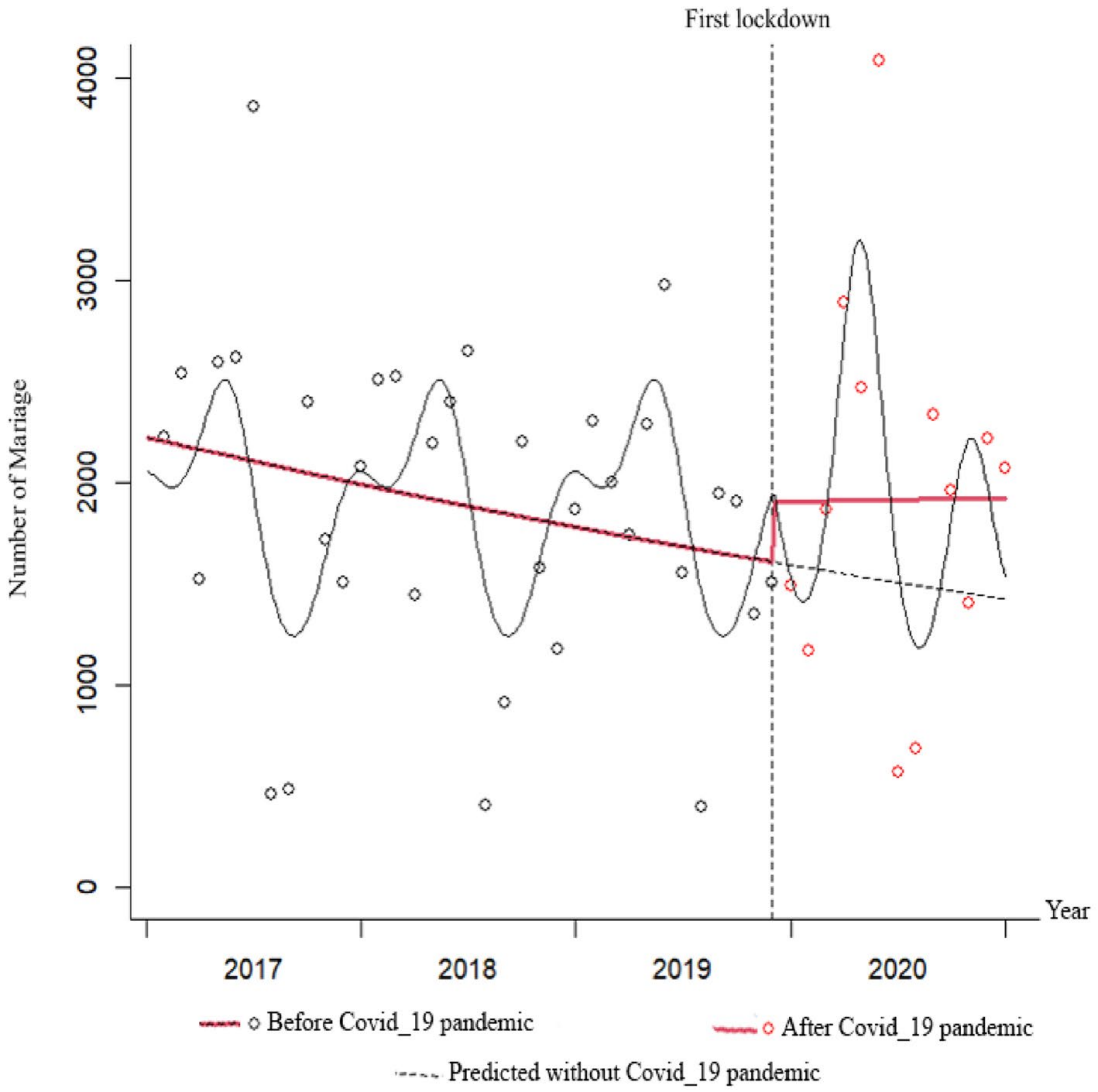
Monthly frequency of marriage before and after the COVID-19 pandemic.

**Figure 6:**
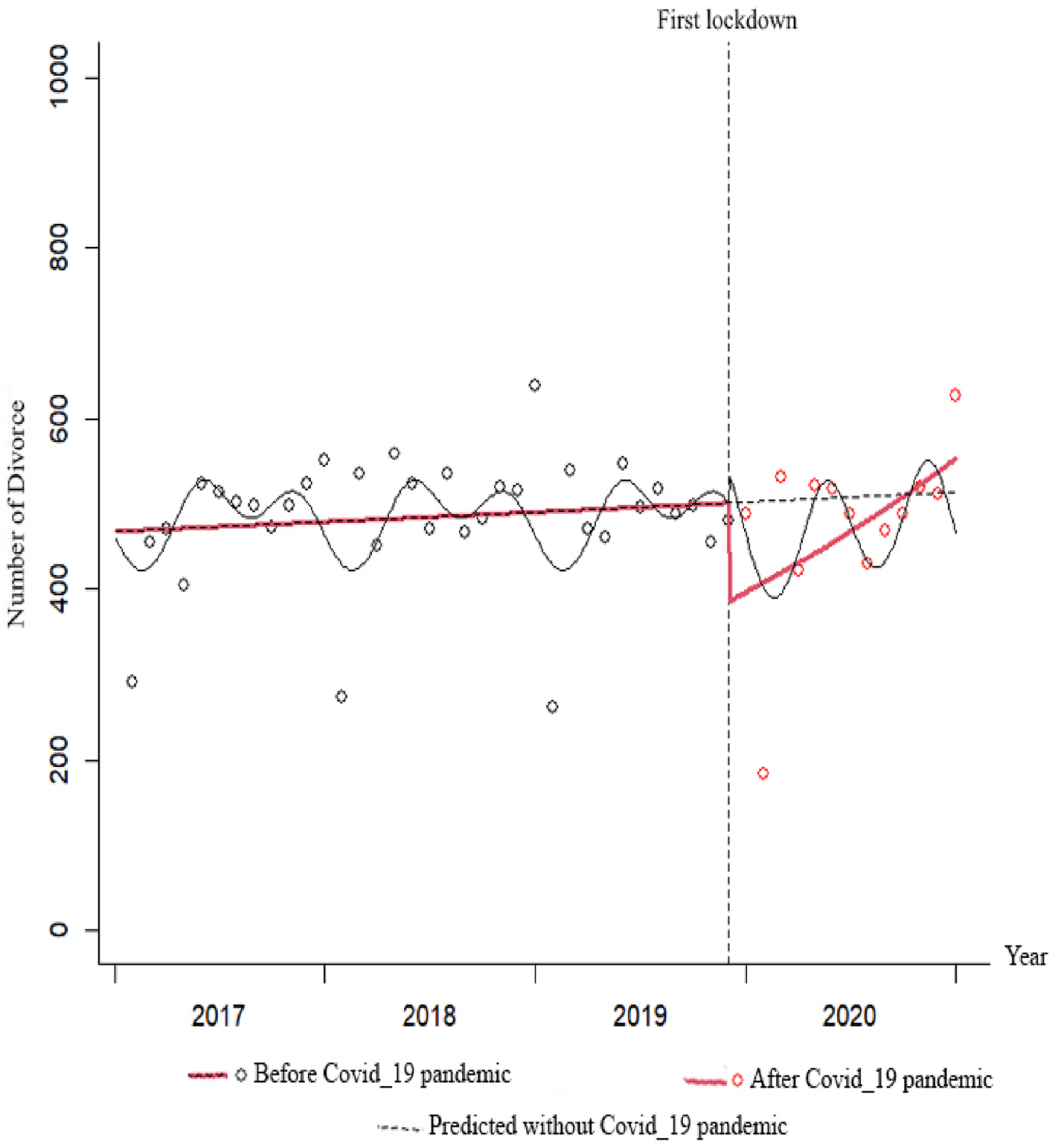
Monthly frequency of divorce before and after the COVID-19 pandemic.

**Figure 7:**
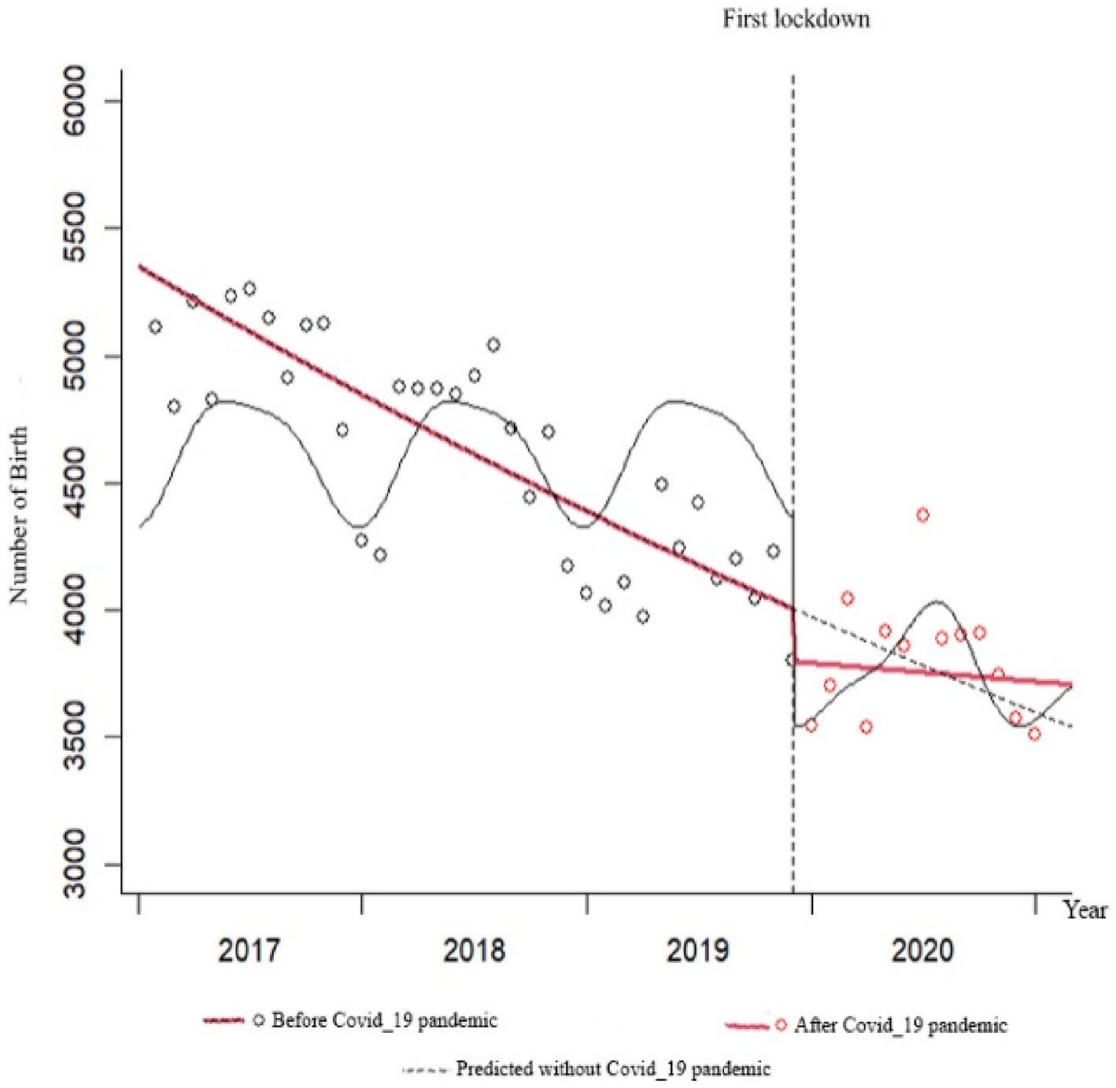
Monthly frequency of birth before and after the COVID-19 pandemic.

**Figure 8:**
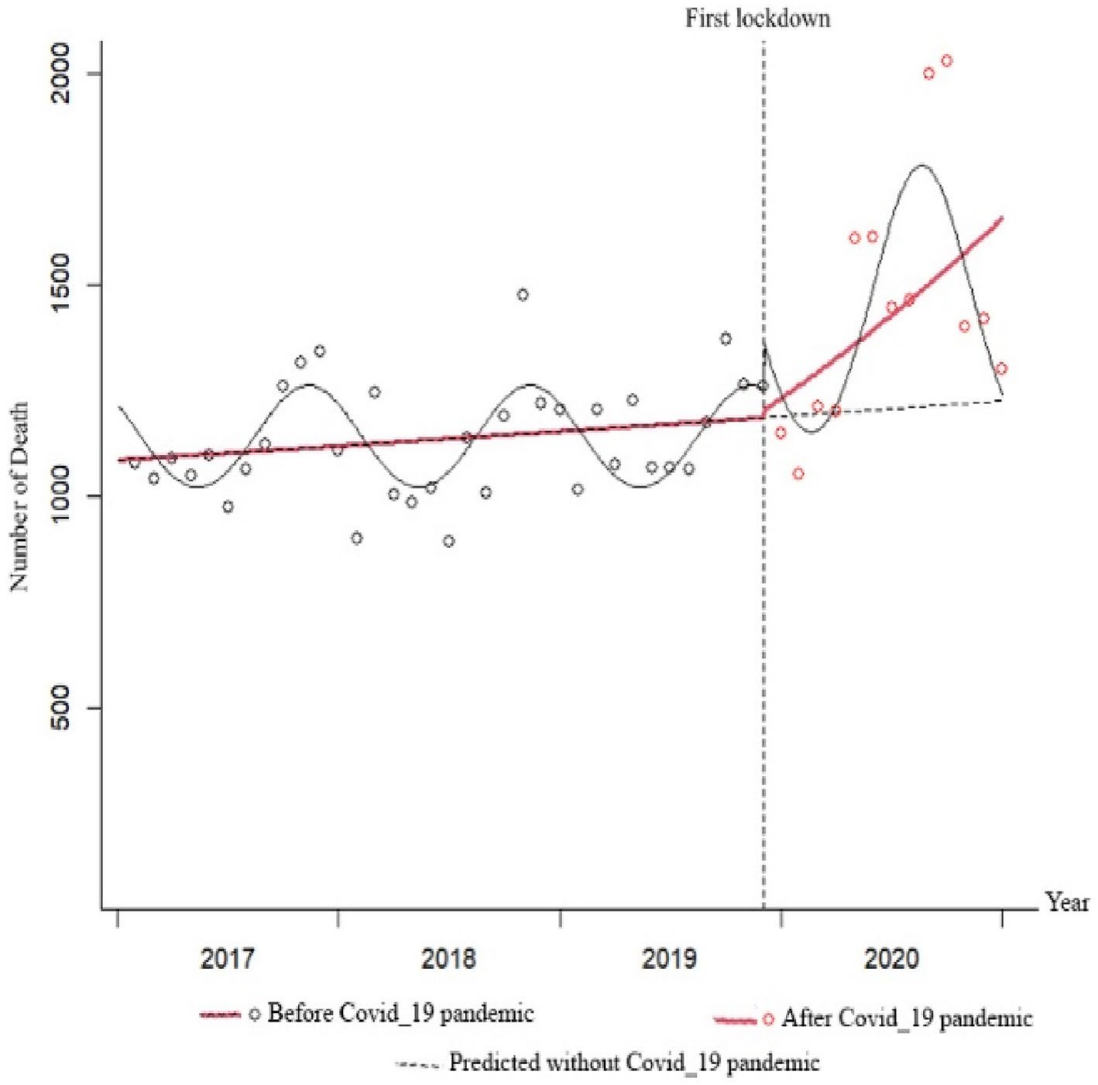
Monthly frequency of death before and after the COVID-19 pandemic.

In terms of births, the trend was sharply decreasing before the pandemic. An immediate decreasing effect was observed at the beginning of the pandemic, followed by a sustained decrease with a slight slope. However, by the end of 2020, the trend of births was higher than predicted based on pre-pandemic data. The trend of death was slightly increasing before the pandemic, and no immediate effect was observed at the beginning of the pandemic. This was followed by a sustained increase with a sharp slope. However, by the end of 2020, the trend of deaths was higher than predicted based on pre-pandemic data (Figs. 5, 6, 7 and 8).

In Figs. 5, 6, 7 and 8, the gray dots show the monthly number of cases before the COVID-19 pandemic, and the red dots show the monthly number of cases during the pandemic. The red dashed line before the first lockdown shows the trend of changes based on the Poisson regression model of the time-series before the pandemic, and the solid red line shows the Poisson trend after the first month of quarantine. The dashed gray line shows the trend of the Poisson regression modeled seasonal pattern based on the data before the COVID-19 lockdown. The slope of the line shows the trend of changes in the number of monthly events.

## Discussion

Our data reveals demographic changes before and after the pandemic. In this study, we utilized aggregated data and were unable to distinguish between individuals belonging to low and high socioeconomic strata. Nevertheless, to gain a deeper understanding of the observed changes in these variables, we deliberated on plausible causes that have been previously discussed in other research works.

A comparison of the number of marriages before and after the COVID-19 pandemic reveals interesting results. The mean number of marriages following the pandemic was higher than before. Before the pandemic, there was a downward trend in marriage rates, but there was an immediate increase in the early stages of the pandemic. After that initial surge, the trend continued to rise, albeit at a slower pace. Finally, we did not observe a significant immediate or sustained effect of the pandemic on the marriage rate. However, the observed trend line of marriages at the end of 2020 was higher than the predicted trend line based on pre-pandemic data. The implementation of social distancing rules to curb the spread of the disease made it challenging to hold extravagant wedding ceremonies. Uncertainty surrounding the duration of the crisis prompted some couples to expedite their marriage registration in order to save on expenses^43^. This factor could explain the observed increase in the marriage rate.

In contrast to our study the United States, Japan, and Italy reported a decrease in marriages during the pandemic^9,11,13^. This decline can be attributed to various factors such as financial difficulties, health concerns, and quarantine measures, which led many couples to postpone or cancel their weddings^9^. During times of economic crisis, individuals, particularly those with lower socioeconomic status, often choose to delay marriage until they have more stability or better employment prospects^13^.

The mean number of divorces following the pandemic was markedly lower than before. Prior to the pandemic, there was a slight upward trend in divorce rates, which experienced an immediate decline at the beginning of the pandemic. However, the trend in divorces subsequently underwent a significant increase during the pandemic. As a result, the observed trend line of divorces at the end of 2020 was higher than the predicted trend line based on pre-pandemic data.

During the early months of the pandemic, divorce rates initially decreased in five US states before rising again in one state^11^. Japan also witnessed a decrease in the number of divorces, particularly during the period when a state of emergency was declared^9^. Conversely, Denmark observed a reduction in divorces in 2020 compared to previous years^14^.

The drop in divorce rates can be attributed to various factors. Fear of infection may have dissuaded individuals from registering their marriages or divorces ^10^. Additionally, the closure of courts and unavailability of legal professionals posed challenges for those seeking to finalize their divorces^44^. The apprehension surrounding extramarital relationships due to infection concerns may have prompted couples to resolve disputes and maintain safer relationships with their spouses. Also during the pandemic adverse macroeconomic circumstances could result in reduced divorce rates. This is because distressed partners may be hesitant to bear the expenses associated with divorces, such as legal fees, court expenses, relocation costs for one or both spouses, the purchase of new furniture, and the division of marital assets^28^.

The increase in the divorce rate can be attributed to the impact of the pandemic on families. The pandemic has brought about changes in employment status and has escalated conflicts and domestic violence within families^10^. Consequently, stress, disputes, conflicts, and ultimately divorces have seen a rise due to the negative consequences of the pandemic^8,9,43^. However, it should be noted that economic factors are the most common cause of divorce^43^.

Iran has experienced a significant surge in divorce rates over the past decade, with economic factors remaining the most common cause of divorce^43^. The rise in housing costs due to a shortage of available housing and limited access to affordable financing, high unemployment rates, and inflation have played a role in driving up divorce rates, particularly among young couples^28^. Moreover, the price of gold in Iran can have an impact on the value of Mehrieh, a customary dowry in Iranian marriages. This increase in value may prompt married women to request their agreed-upon Mehrieh earlier, potentially leading to heightened family conflicts and a higher divorce rate^45^. Reasons for divorce in Iran include infidelity, extramarital affairs, sexual dissatisfaction, addiction-related domestic violence, and women's empowerment, including increased independence, higher education levels, and advocacy for their basic rights. Desperation, anger, unemployment, and poverty contribute to the vulnerability of individuals to violence. Quarantine policies further exacerbate stress, financial insecurity, isolation, limited personal space, and poor social and economic status, all negatively impacting mental health. As a result, the pandemic has led to an increase in domestic violence and divorces in Iran^46^.

Previous studies suggest various interventions that can help reduce disputes and divorce rates. Engaging in religious practices such as family prayer, studying religious texts, and worship can strengthen positive family interactions during the pandemic^47^. Additionally, individual or group spiritual practices like yoga, meditation, mindfulness, and enjoying nature with family have been found to be effective^48^. Furthermore, online training and seminars focused on family topics can be conducted to prevent family disputes and raise public awareness about divorce^49^.

The COVID-19 pandemic has impacted birth rates in Kerman province. Although the mean number of births decreased following the pandemic, this difference was not statistically significant. Before the pandemic, there was a downward trend in births that immediately declined at its onset. Post-pandemic, the decline continued but at a slower rate than pre-pandemic levels. Interestingly, the observed trend line of births at the end of 2020 was higher than the predicted trend line based on pre-pandemic data.

The study's findings align with research conducted in other countries, such as New York, Italy, Japan, and China, which have also reported a decrease in birth rates during the COVID-19 pandemic^9,16–18^. It is important to note that pandemics can initially affect fertility intentions, but the actual impact on birth rates occurs with a delay. This is because the birth rate during the initial months of the pandemic is determined by decisions made by individuals approximately nine months earlier. Before the pandemic, fertility rates had already been declining in both high-income and low-income countries^26^. On the other hand, the decision to have children is influenced by cultural, social, economic, and political considerations within a particular society^50^.

Economic pressure and reduced income were identified as significant influencing factors that deterred couples from having children^18^. Uncertainty in the labor market, general societal uncertainty, and negative expectations about the future also contributed to the decline in fertility rates^15^. In Iran, the decline in fertility rates is linked to women's educational attainment, the timing of marriage and family formation, family planning and contraceptive use, economic insecurity, the influence of social networks, and changing attitudes toward marriage and fertility^51^. The negative impact of the pandemic on birth rates can be attributed to various factors, including challenges faced by housewives during quarantine, fear, anxiety, personal health problems, internet addiction, and poor mental health^46^. Consequently, couples altered their intentions regarding childbearing during this period^9^. Furthermore, the impact of migration to less densely populated areas on birth registration remains uncertain^16^. Therefore, further research is necessary to understand how social and demographic factors influence birth rates during pandemics^17^.

There was a significant rise in the rate of deaths following the pandemic. Prior to the pandemic, there was a slight upward trend in mortality rates, which was exacerbated during the pandemic. While the immediate impacts of the pandemic on mortality rates were not observed, there was a sustained effect over time. However, the observed trend line of deaths at the end of 2020 was higher than the predicted trend line based on pre-pandemic data.

Similar observations of increased death rates during the pandemic have been reported in Iran and the United States^19,20^. In Colombia, non-COVID-19 deaths increased in the five states with the highest COVID-19 death tolls, which may be attributable to undetected or unrecorded COVID-19-related infection^21^.

The outbreak of COVID-19 in Iran has caused a significant economic setback, which coincided with the imposition of severe political sanctions. The healthcare system, which was already grappling with numerous challenges, was severely impacted by these sanctions, leading to a shortage of crucial medical resources. As a result, the healthcare sector faced significant hurdles in delivering adequate care to patients, which further contributed to the loss of lives among Iranians due to the virus^52^. Under normal circumstances, death is not directly influenced by decision-making processes like marriage, divorce, and birth. However, during the pandemic, the increased mortality rate can be partially attributed to individuals contracting illnesses other than COVID-19 or having pre-existing diseases. Many refrained from seeking medical attention due to fear of contracting and dying from COVID-19, which ultimately resulted in more fatalities^53^. Therefore, it is evident that individual choices can have an impact on mortality rates. Thus, it is crucial to continue promoting preventive measures such as mask-wearing, hand hygiene, and social distancing to curb the spread of the virus. Additionally, vaccination and the implementation of comprehensive screening systems for rapid diagnosis, adequate treatment, and provision of necessary medications are also essential in reducing mortality rates.

The COVID-19 pandemic has led to negative population growth in Kerman province, which can accelerate population aging and have adverse effects on the economy, healthcare system, and social well-being. Policymakers need to improve health and population policies to address population aging, drawing lessons from the experiences of Western countries^54^. Population growth in Iran also exacerbates environmental pollution, so energy consumption and population development policies should align with environmental efforts^55^. International economic sanctions have had negative effects on Iranians' health status, leading to increased living costs, unemployment, and limited access to essential medicines^56^. Given the consequences of negative population growth, the impact of population increase on mental health, and the problems caused by international sanctions, necessary measures should be taken to increase the population.

The study found that there were higher frequencies of birth and death in men compared to women, with higher sex ratios at birth and death for males in all four years. The COVID-19 pandemic has led to increased death rates among men, both globally and in specific countries like Massachusetts, Sweden, and China^57–60^. The higher mortality in men may be influenced by social factors such as smoking and lower handwashing rates, as well as biological factors related to sex hormones 57. Women generally have stronger immune responses, potentially due to genetic factors. Men also have more angiotensin-converting enzyme 2 (ACE2), receptors, making them more susceptible to the virus. Smoking, which is more common among men, can further increase vulnerability to COVID-19 ^60^. Understanding mortality rates in different age and gender groups can provide important insights for targeted interventions.

## Conclusion

The COVID-19 pandemic has had far-reaching effects on marriage and divorce rates, birth rates, and death rates. Quarantine measures and fear of the virus influenced people's decisions, resulting in changes in these demographic indicators. While marriage and divorce rates were immediately affected, birth rates experienced a time lag. This can be attributed to the time lag between decisions regarding starting to have children and the actual births. The pandemic has led to an increase in death rates, with individuals avoiding medical attention due to COVID-19 fears. Vaccination and effective treatment strategies are crucial in mitigating this impact. Financial aid and support for families are important due to the role of economic problems in couples' decisions. It is vital to monitor these indicators and implement strategies that prioritize public health and well-being.

### Limitations and suggests

The study has several limitations. Firstly, being an ecological study, it cannot establish a strong causal relationship between variables, but it can generate hypotheses. Secondly, confounding variables were not controlled, making it difficult to determine true cause and effect. Additionally, the use of aggregated data prevented differentiation between COVID-19 and non-COVID-19 deaths, limiting the understanding of the disease’s impact on mortality rates. Future studies should focus on collecting detailed data to separate COVID-19 and non-COVID-19 deaths for a more comprehensive analysis. It is also recommended to investigate reasons for marriage and divorce post-pandemic and analyze marriage, divorce, birth, and death rates in the second year after the pandemic to determine if trends persist or change over time.

## Data Availability

The data presented in this study are available on request from the corresponding author.
